# Sulphide of Calcium in Scrofulous Abscesses and Boils

**Published:** 1875-07

**Authors:** T. Curtis Smith

**Affiliations:** Midleport, Ohio


### SULPHIDE OF CALCIUM IN SCROFULOUS ABSCESSES AND BOILS.

BY T. CURTIS SMITH, M. D., MIDLEPORT, OHIO.

No physician of experience need be told of the great annoyance, caused to himself and his patients, that these affections produce.—A scrofulous abscess occurring in a child that has the well-marked diathesis is very apt to be of long duration. It is slow in forming, being first of the appearance of an enlarged and indurated gland, gradually increasing in size and hardness till it reaches its utmost limit of swelling before any sign of softening appears. This process often occupies weeks, sometimes months. Then it may require a period of the same length for it to soften and give indications of opening spontaneously. When once opened, whether by nature or the lance of the surgeon, another long period may elapse before the abscess is entirely healed, and the tissues affected assume their natural appearance and color. It is true that all these cases are not thus prolonged, but many of them are, and occassionally proves fatal. Such instances are not very rare in the hands of able practitioners, under the best of treatment. As to boils, we not infrequently meet with cases where there is a succession of crops, one crop going through the process of recession while the next crop is coming on. These, though seldom, if ever, proving fatal, produce the greatest annoyance and suffering to the patient and often tax the ability of the physician in securing the needed relief. Such being the facts, it is not surprising that we should look for anything that gives fair promise of speedy relief, or that we resort to use even though we do not thoroughly comprehend the method of its action on the physical economy.

The two following cases will probably illustrate the value of the sulphide of calcium in the kind of cases above named :

J. C., æt. 2 years, mulatto, of a highly scrofulous temperament, always gives the appearance of a sickly child. It is badly surrounded, the place of its residence on low ground, is filthy both in and outside the house. In December, 1874, the child was attacked with diarrhœa, which greatly reduced its strength and flesh, but from which it recovered after a week of careful treatment. Immediately following the above attack and before the child's strength had much recuperated, a swelling appeared under and posterior to the chin, in the median line or centrally. This came on with more rapidity than such abscesses commonly do, owing, no doubt, to the reduced state of the child's health. It was placed upon the use of ferri iodid. in twenty-drop doses four times a day and the liberal use of wine and milk, with beef essence, bread, etc. After a week it seemed to stop swelling and remain hard and gave indications of being a long time in forming an abscess, which it would evidently do some time in the future. The child's feeble condition, considered with its very unfavorable hygienic surroundings, led me to no very happy conclusion as to the final result of the case. Adding to or strengthening this conclusion, was the weight of a renewed attack of diarrhœa and rejection of almost everything given it, whether medicine or food. Stopping all other medicines, but dieting the child as carefully as I could, and giving it as much pure air as the inclemency of the weather at that season would permit, I gave it in glycerin the one-tenth grain of sulphide of calcium every three hours. Happily it did not reject this, and after a day the dose was gradually increased to one-third of a grain every three hours. The diarrhœa ceased the third day after commencing its use; but this I did not attribute to the sulphide, believing it to be accidental or due to the course of diet pursued, and to the additional fresh air and clean garments it received. In less than forty-eight hours after the first dose of the sulphide of calcium was given, the abscess showed evident signs of softening, and on the fourth day burst spontaneously, discharging very profusely a semi-laudable pus. The suppuration was more profuse than I had expected to see in this instance, but soon began to diminish.—Healthy granulations now put in appearance. These continued until the abscess entirely disappeared and healing was completed, which from the time of opening occupied the two weeks. As the healing process went on and approached completion, the child's strength and flesh gradually increased, its appetite became very ravenous and digestion good. It is needless to say that its suffering from the abscess was intense, or to mention the many irritative symptoms naturally attending the process of its painful formation, as these are readily remembered by every one who has attended cases of this character.

I was agreeably surprised at the speedy resolution of the swelling, and still more so at its ready and rapid recovery after the very free suppuration in a patient as feeble as was this child. Under the iron and a tonic alterative course such cases do not usually recover under one, more frequently two or three months.

*Boils.*—Mrs. A——, an old lady, after being afflicted for some weeks with a great degree of general debility following an attack of muscular rheumatism, was the unfortunate victim of several boils, which appeared upon the back and the left shoulder. These were very painful and of the kind vulgarly known as "blood-boils." She was unable to lay on the back or left side, and confinement to the right side became very irksome. In time these began to disappear, having suppurated very little, though they were well poulticed and opened with a lance. As their retrocession began, a new crop put in an appearance. I now placed her on grain doses of the sulphide of calcium every four hours. The new crop of furuncles continued to advance for the next twenty-four hours, but no longer. There was then a short period, during which no change was observable. But soon they began to diminish in color and size, and gradually disappeared without suppurating.

The first crop of furuncles, meanwhile, began to discharge pus freely, which continued for two or three days, after which they very rapidly disappeared. Two cases prove but very little, but the action of the agent seemed to be so favorable in these two cases, which I think are perfectly typical, that I give them to the profession for what they may be worth.

Other cases could be related where I have resorted to the agent with appparent good success, but not as well marked as in those above related. They were not, however, of the same class of cases. I am led to believe from the limited use I have made of the agent that it will prove a very valuable weapon with which to combat inflammations anywhere, that tend to end in suppuration. Further observation, however, is necessary to either confirm or disprove this opinion.

This plan of treatment is not original with me, as it has already been set forth by Sidney Ringer and others through the medical press. These writers however do not restrict themselves to the sulphide of calcium, but include also the sulphides of sodium and potassium, which are quite as efficacious in the treatment of suppurative inflammations.

